# A systematic review of interventions to improve prevention of mother-to-child HIV transmission service delivery and promote retention

**DOI:** 10.7448/IAS.19.1.20309

**Published:** 2016-04-06

**Authors:** Julie Ambia, Justin Mandala

**Affiliations:** 1 KAVI-Institute of Clinical Research, University of Nairobi, Nairobi, Kenya; 2 Department of Global Health, Population, and Nutrition, FHI 360, Washington, DC, USA

**Keywords:** infant ART initiation, conditional cash transfer, male involvement, peer mentoring, community health worker, home visit, mobile phone-based reminders, integrated PMTCT services

## Abstract

**Introduction:**

The success of prevention of mother-to-child transmission of HIV (PMTCT) is dependent upon high retention of mother-infant pairs within these programmes. This is a systematic review to evaluate the effectiveness of interventions that aim to improve PMTCT service delivery and promote retention throughout the PMTCT steps.

**Methods:**

Selected databases were searched for studies published in English (up to September 2015). Outcomes of interest included antiretroviral (ARV) drugs or antiretroviral therapy (ART) initiation among HIV-positive pregnant and/or breastfeeding women and their infants, retention into PMTCT programs, the uptake of early infant diagnosis (EID) of HIV and infant HIV status. Risk ratios and random-effect meta-analysis were used in the analysis.

**Results:**

Interventions assessed in the 34 identified studies included male partner involvement in PMTCT, peer mentoring, the use of community health workers (CHWs), mobile phone-based reminders, conditional cash transfer, training of midwives, integration of PMTCT services and enhanced referral. Five studies (two randomized) that evaluated mobile phone-based interventions showed a statistically significant increase (pooled RR 1.18; 95% CI 1.05 to 1.32, *I*^2^=83%) in uptake of EID of HIV at around six weeks postpartum. Male partner involvement in PMTCT was associated with reductions in infant HIV transmission (pooled RR 0.61; 95% CI 0.39 to 0.94, *I*^2^=0%) in four studies (one randomized). Four studies (three randomized) that were grounded on psychological interventions reported non-significant results (pooled RR 1.01; 95% CI 0.93 to 1.09, *I*^2^=69%) in increasing ARV/ART uptake among HIV-positive pregnant and/or breastfeeding women and infant HIV testing (pooled RR 1.00; 95% CI 0.94 to 1.07, *I*^2^=45%). The effect of the other interventions on the effectiveness of improving PMTCT uptake was unclear. Heterogeneity of interventions limits these findings.

**Conclusions:**

Our findings indicate that mobile phone-based reminders may increase the uptake of EID of HIV. Studies on male partner involvement in PMTCT reported reductions in infant HIV transmission. Stronger evidence is needed and future studies should determine the long-term effects of these interventions in improving retention throughout the PMTCT steps.

## Introduction

To fully benefit from the prevention of mother-to-child transmission of HIV (PMTCT), HIV-positive pregnant and/or breastfeeding women and their infants must successfully navigate a number of steps. These steps, also referred to us the PMTCT cascade, include maternal HIV testing; and for HIV-positive mothers, assessment of treatment eligibility, initiation of maternal antiretroviral (ARV) drugs or antiretroviral therapy (ART), initiation of infant ARV, infant HIV testing and ART initiation for HIV-infected infants [1]. Approximately 90% retention is required at each step to effectively reduce mother-to-child transmission of HIV (MTCT) [2]. However, loss to follow-up occurs at all stages of the PMTCT cascade [1]. A systematic review of 44 studies in sub-Saharan Africa showed that 94% of pregnant women were tested for HIV, 70% of those who were HIV-positive initiated ARV/ART, 64% of the HIV-exposed infants (HEIs) were tested for HIV at six weeks and 55% of these infants received their final diagnosis at 18 months [3].

Recent efforts to increase PMTCT utilization include health-systems strengthening, enhanced counselling, community/partner support and educational strategies [4]. However, to our knowledge, these interventions have not been synthesized in a comprehensive review. We therefore undertook a systematic review to evaluate the effectiveness of these interventions aimed at improving service delivery and promoting retention along the PMTCT cascade.

## Methods

### Inclusion and exclusion criteria

Studies of interest were those that evaluated interventions to improve service delivery and promote retention in the following steps of PMTCT services:

1. Initiation of ARV/ART among pregnant and/or breastfeeding women
2. Uptake of early infant HIV testing
3. Early initiation of ART among HIV-infected infants

We selected randomized controlled studies and non-randomized controlled studies from any part of the world. Participants were HIV-positive pregnant women and mother-infant pairs accessing PMTCT services.

Studies were excluded if the intervention(s) had no comparison group, was a quality improvement program and evaluated a structural intervention that was not implemented in a health facility, for example, community sensitization campaigns.

### Data extraction

One reviewer independently screened full articles and conference abstracts that appeared to meet the search criteria. Using a standardized data extraction form, one reviewer recorded the data for included studies that included author, study period, study country, study design, follow-up period, and details of intervention and control conditions. A random sample of 50% of the extraction was confirmed by a second reviewer. Uncertainties were resolved through discussions between the two reviewers.

### Search strategies

We searched PubMed, Web of Science, Embase and ClinicalTrials.gov databases and abstracts of the International Conference on AIDS and STIs in Africa, the International AIDS Society Conference on HIV Pathogenesis and Treatment, and the Conference on Retroviruses and Opportunistic Infections up to September 2015. Reference lists from reviews and included studies were also searched. Only studies published in English were included. The search strategies included a combination of the following key terms: HIV, ART, PMTCT, pregnant and utilization (Supplementary file 1).

### Quality assessment

Quality assessment was guided by Effective Public Health Practice Project criteria. We rated each of the quality components in terms of selection bias, study design, confounders, blinding, data collection methods, withdrawals/dropouts and integrity of intervention. Reviewers rated each component as strong, moderate, or weak.

### Data analysis

Each study results were expressed as a risk ratio (RR) with its 95% confidence intervals (CIs). When studies included multiple time points, the RR was calculated for each outcome. Random effects meta-analysis was used to give pooled estimates if two or more studies had the same intervention and reported the same outcome. Heterogeneity among studies was examined using both the *χ*^2^ test and the I^2^ statistic.

When multiple data sets were provided for the number of HEIs tested, data were extracted for the collection of results from the facility by the mother. When there were no events in the control or intervention group, 0.5 was added to each cell of the 2×2 table. A baseline sample size was used if the authors did not specify the number of participants involved in the final analysis assessing the outcomes of intervention. We used the RevMan 5.3, and Practical Meta-Analysis Effect Size Calculator (www.campbellcollaboration.org/resources/effect_size_input.php) to conduct all analyses.

## Results

### Study selection and characteristics

**Figure 1:**
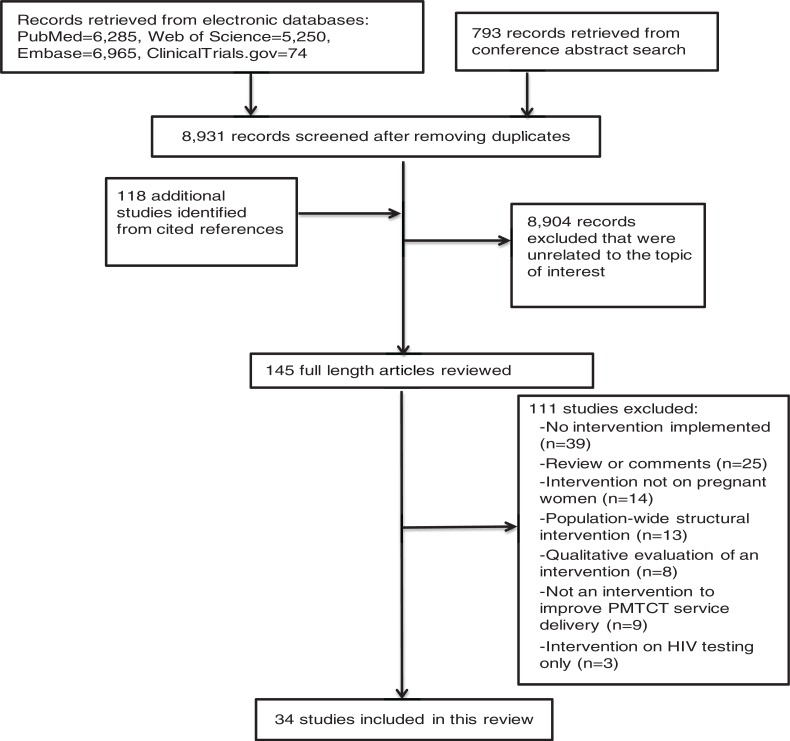
Flow chart of studies included in these review.

The search results are summarized in Figure 1. Thirty-four studies were included in this review (Supplementary file 2). Total sample size of mother-infant pairs ranged from 30 to 7875. Twenty-one studies aimed to improve ARV/ART initiation of HIV-positive pregnant and/or breastfeeding women (Table 1), eight studies aimed to improve retention in PMTCT programmes (Table 2), 19 studies aimed to increase uptake of early infant diagnosis (EID) (Table 3) and two studies aimed to improve early initiation of infant ART (Table 4). Fourteen studies reported infant HIV status (Table 5). Most studies measured several outcomes on the PMTCT cascade. Most studies were conducted in sub-Saharan Africa: 13 in South Africa [5–17]; eight in Kenya [18–25]; two studies each in Malawi [26,27], Zambia [28,29] and Mozambique [30,31]; and one study each in Democratic Republic of Congo [32], Swaziland [33], Tanzania [34], Rwanda [35] and Ethiopia [36]. Only one study was conducted in United States of America [37].

**Table 1:** Evaluation of interventions to increase initiation of ARV/ART in pregnant women

			Mother initiated on any antenatal treatment regimen of ARV/ART/ HIV+ pregnant women		Effect size (95% CI) for MTCT outcome		
				
Intervention category	Summary of intervention type	Author	Intervention	Control	RR	Lower 95% CI	Upper 95% CI
Social	Peer mentoring	ENHAT-CS 2014 [36]	373/583 (64%)	147/294 (50%)	**1.28**	1.12	1.46
		Baek 2007 [13]	116/125 (93%)	92/111 (3%)	**1.12**	1.02	1.23
		Futterman 2010 [15]	31/31 (100%)	38/40 (95.0%)	1.05	0.96	1.15
		Richter 2014 [5]	340/377 (90.2%)	445/466 (95.5%)	0.94	0.91	0.98
	CHWs	Kim 2012 [26]	526/1318 (40%)	1284/14,669 (8.8%)	**4.56**	4.19	4.96
		le Roux 2013 [12]	169/185 (91.4%)	149/169 (88.2%)	1.04	0.97	1.11
	Male partner involvement	Msuya 2008 [34]	29/32 (90.6%)	101/137 (73.7%)	**1.23**	1.06	1.43
		Aluisio 2011 [23]	133/140 (95%)	294/316 (93%)	1.02	0.97	1.07
		Kalembo 2013 [27]	50/60 (83%)	76/102 (74.5%)	1.18	0.95	1.31
		Weiss 2013 [17]	9/12 (75%)	6/12 (50%)	1.5	0.78	2.88
Behavioural	Mobile phone text messages	Finocchario-Kessler 2014 [22]	462/523 (88%)	251/320 (78%)	**1.13**	1.06	1.20
Structural	Training of midwives	Kieffer 2011 [33]	369/459 (80%)	320/463 (69%)	**1.16**	1.08	1.25
	Enhanced referral	Ciampa 2011 [30]	15/63 (23.0%)	47/332 (14.0%)	1.68	1.00	2.82
	Integration of PMTCT into routine pregnancy and infant care	Killam 2010 [28]	376/846 (44.4%)	181/716 (25.3%)	**1.76**	1.52	2.04
		Stinson 2010 [9]	183/227 (80.6%)	99/130 (76.2%)	1.06	0.94	1.19
		Stinson 2013 [10]	120/215 (55.8%)	70/155 (45.2%)	1.24	1.00	1.53
		Tsague 2010 [35]	511/532 (96%)	106/106 (100%)	0.96	0.94	0.99
		Turan 2015 [18]	528/569 (93%)	581/603 (96%)	0.96	0.94	0.99
		van't Hoog 2005 [24]	356/625 (57%)	369/683 (54%)	1.05	0.96	1.16
	Integration of public health services into clinical care for HIV-positive pregnant women and HEIs	Ezeanolue 2015 [37]	85/105 (81%)	16/26 (61.5%)	1.32	0.96	1.81
Social and structural	Integration of PMTCT and ANC services, lab courier system for CD4 counts and use of lay counsellors	Herlihy 2015 [29]	133/186 (71.5%)	38/138 (27.5%)	**2.6**	1.95	3.45

The RR values of studies that showed evidence of association are given in bold.

**Table 2:** Evaluation of interventions to improve retention in PMTCT services

			Completed follow-up of mother-infant pairs			Effect Size (95% CI) for retention outcome		
					
Intervention category	Summary of intervention type	Author	Intervention	Control	Follow-up period	RR	Lower 95% CI	Upper 95% CI
Social	Male partner involvement	Kalembo 2013 [27]	47/54 (87%)	55/407 (13.4%)	18 months	**6.44**	4.93	8.41
		Weiss 2013 [17]	30/30 (100%)	39/39 (100%)	6 weeks	1	1	1
	Peer mentoring	Futterman 2010 [15]	23/40 (57.5%)	11/31 (35.5%)	6 months	1.62	0.94	2.79
Behavioural	Mobile phone text messages	Finocchario-Kessler 2014 [22]	488/523 (93.3%)	156/320 (48.8%)	18 months	**1.91**	1.71	2.15
		Odeny 2014 [19]	38/194 (19.6%)	22/187 (11.8%)	8 weeks	**1.66**	1.03	2.70
	Mobil-phone calls	Kebaya 2015 [25]	68/75 (90.7%)	54/75 (72%)	6 weeks	**1.26**	1.07	1.48
	Conditional cash transfers	Yotebieng 2015 [32]	167/216 (77.3%)	149/217 (68.7%)	6 weeks	1.13	1.00	1.26
Structural	Integration of PMTCT into routine pregnancy and infant care	Killam 2010 [28]	244/278 (87.8%)	94/103 (91.3%)	3 months	0.96	0.89	1.04

The RR values of studies that showed evidence of association are given in bold.

**Table 3:** Evaluation of interventions to improve uptake of EID

			Infants tested/HEIs		Effect size (95% CI) for uptake of EID outcome		
				
Intervention category	Summary of intervention type	Author	Intervention	Control	Follow-up period (age of infant testing)	RR	Lower 95% CI	Upper 95% CI
Social	Peer mentoring	ENHAT-CS 2014 [36]	466/583 (80%)	212/294 (72%)	≤2 months	**1.11**	1.02	1.20
		ENHAT-CS 2014 [36]	35/583 (6%)	0/294 (0%)	18 months	**35.86**	2.21	582.60
		Shroufi 2013 [38]	121/122 (99.2%)	17/35 (48.6%)	6 to 8 weeks	**2.04**	1.45	2.87
		Futterman 2010 [15]	34/40 (85%)	30/31(96.8%)	6 months	0.88	0.76	1.02
		Rotheram-Borus 2014 [6]	206/284 (72.5%)	247/344 (71.8%)	6 weeks or 6 months	1.01	0.92	1.11
	CHWs	Kim 2012 [26]	1064/1318 (80.7%)	7875/14,669 (53.7%)	≤3 months	**1.50**	1.46	1.55
		Tomlinson 2014 [14]	420/571 (73.6%)	465/698 (66.6%)	6 weeks	**1.10**	1.03	1.19
		le Roux 2013 [12]	155/185 (96.9%)	132/169 (94.3%)	6 weeks	1.07	0.97	1.19
	Patient advocates	Rundare 2012 [7]	710/2781 (25.5%)	322/1356 (23.7%)	6 weeks	1.08	0.96	1.21
	Male involvement	Msuya 2008 [34]	21/26 (80.8%)	74/111 (66.7%)	18 months	1.21	0.96	1.52
		Weiss 2013 [17]	30/30 (100%)	39/39 (100%)	6 weeks	1	1	1
Behavioural	Calls and mobile phone text messages	Schwartz 2015 [8]	45/50 (90%)	32/50 (63.3%)	10 weeks	**1.41**	1.12	1.77
	Mobile phone text messages	Finocchario-Kessler 2014 [22]	523/523 (100%)	242/320 (75.6%)	6 weeks	**1.32**	1.24	1.41
		Finocchario-Kessler 2014 [22]	137/166 (82.5%)	62/168 (36.9%)	9 months	**2.24**	1.81	2.76
		Joseph-Davey 2013 [31]	201/261 (77.1%)	185/261 (70.9%)	8 weeks	1.09	0.98	1.20
		Technau 2011 [11]	108/160 (67.3%)	110/177 (61.9%)	10 weeks	1.09	0.93	1.27
		Odeny 2014 [19]	172/187 (92.0%)	154/181 (85.1%)	8 weeks	1.08	1.00	1.16
Structural	Enhanced referral	Ciampa 2011 [30]	34/63 (54.0%)	85/332 (25.6%)	≤3 months	**2.11**	1.57	2.82
	Integration of PMTCT into routine pregnancy and infant care	Washington 2015 [40]	143/568 (25%)	106/594 (17.8%)	6 weeks	**1.41**	1.13	1.76
		Turan 2015 [18]	361/569 (63.4%)	326/603 (54.1%)	9 months	**1.17**	1.07	1.29
Structural and social	Integration of PMTCT into routine pregnancy and infant care and use of peer counsellors	Ong'ech 2012 [20]	109/179 (60.9%)	84/184 (45.7%)	12 months	**1.33**	1.10	1.62
		Ong'ech 2012 [20]	177/178 (98.9%)	182/182 (100%)	6 to 8 weeks	0.99	0.98	1.01
	Integration of PMTCT and ANC services, lab courier system for CD4 counts and use of lay counsellors	Herlihy 2015 [29]	309/553 (55.8%)	212/506 (41.9%)	6 weeks	**1.33**	1.18	1.51
	Integration of PMTCT and ANC services, lab courier system for CD4 counts and use of lay counsellors	Herlihy 2015 [29]	436/553 (78.8%)	347/506 (68.9%)	12 months	**1.15**	1.07	1.24
Social and behavioural	Peer mentoring and mobile phone calls	Besser 2010 [16]	167/214 (78%)	114/204 (56%)	16 weeks	**1.40**	1.21	1.61

The RR values of studies that showed evidence of association are given in bold.

**Table 4:** Evaluation of interventions to increase early antiretroviral therapy initiation among HIV-infected infants

			HIV-infected infants initiated on ART		Effect size (95% CI) for infant ART outcome		
				
Intervention category	Summary of intervention type	Author	Intervention	Control	RR	Lower 95% CI	Upper 95% CI
Social	CHWs	Kim 2012 [26]	33/43 (76.7%)	110/320 (34.4%)	**2.23**	1.79	2.79
Behavioural	Mobile phone text messages	Finocchario-Kessler 2014 [22]	22/22 (100%)	10/21 (47.6%)	**2.1**	1.34	3.29

The RR values of studies that showed evidence of association are given in bold.

**Table 5:** Evaluation of infant HIV status

			HIV-infected infants/HEIs tested		Effect size (95% CI) for MTCT outcome		
				
Intervention category	Summary of intervention type	Author	Intervention	Control	FU period for infant testing	RR	Lower 95% CI	Upper 95% CI
Social	Male partner involvement	Aluisio 2011 [23]	17/140	65/316	12 months	**0.59**	0.36	0.97
		Farquhar 2004 [21]	1/9 (11%)	7/58 (12%)	3 months	0.92	0.13	6.63
		Kalembo 2013 [27]	4/47 (8.5%)	7/55 (12.7%)	18 months	0.67	0.21	2.14
		Weiss 2013 [17]	1/30 (3%)	3/39 (8%)	6 weeks	0.43	0.05	3.96
	Peer mentoring	ENHAT-CS 2014 [36]	11/ 210 (5.2%)	31/ 272 (11.4%)	2 to 12 months	**0.46**	0.24	0.89
		Rotheram-Borus 2014 [6]	7/284 (2.6%)	9/344 (2.5%)	6 weeks or 6 months	0.94	0.36	2.50
		Shroufi 2013 [38]	1/34 (2.9%)	0/121 (0%)	6 to 8 weeks	10.46	0.44	251.07
	CHWs	Kim 2012 [26]	43/1047 (4.1%)	1084/7875 (13.8%)	≤3 months	**0.30**	0.22	0.40
		Tomlinson 2014 [14]	37/580 (6.4%)	47/714 (6.6%)	12 weeks	0.97	0.64	1.47
	Patient advocates	Rundare 2012 [7]	3/710 (0.4%)	16/322 (5.0%)	6 weeks	**0.09**	0.03	0.29
Behavioural	Mobile phone text messages	Odeny 2014 [19]	2/172 (1.2%)	3/154 (1.9%)	8 weeks	0.60	0.10	3.53
Structural	Integration of PMTCT into routine pregnancy and infant care	Washington 2015 [40]	6/143 (4.2%)	7/106 (6.6%)	6 weeks	0.64	0.22	1.84
		Washington 2015 [40]	28/382 (7.3%)	27/338 (8%)	9 months	0.92	0.55	1.53
	Integration of public health services into clinical care for HIV-positive pregnant women and HEIs	Ezeanolue 2015 [37]	0/105 (0%)	6/26 (23.1%)	Not reported	**0.02**	0.00	0.34

The RR values of studies that showed evidence of association are given in bold.

### Interventions

Eight interventions from 34 studies were included in this review. The primary groupings of the interventions were social, behavioural and structural.

#### Social interventions

Fifteen studies were based on social interventions. Social interventions were defined as any form of social support coming from the male partner, family, peers and community health workers (CHWs). Seven studies examined the effectiveness of peer mentoring interventions as compared to regular/routine PMTCT services [5,6,13,15,16,36,38]. Peer mentoring interventions involved mentor mothers who were also HIV-positive, had had a child recently, had used PMTCT services and were coping positively. Three of the peer mentoring interventions employed psychological interventions, such as a cognitive-behavioural approach [5,6,15]. Group and individual sessions were held by the peer mentors in the seven studies. With regards to timing, six out of seven studies implemented interventions during antenatal and postnatal period. One did not report the timing of interventions [16]. Four of the peer mentoring interventions followed up mother-infant pairs at home and health facilities [13,16,36,38]; one of these studies made phone calls to mothers who had missed their EID appointments [16].

Five studies evaluated the association of male partner involvement in PMTCT utilization [17,21,23,27,34]. Four studies sought to determine whether male partner involvement, described as a male partner accompanying his pregnant spouse to antenatal clinic, would impact infant HIV acquisition [23] and PMTCT uptake [21,27,34]. The other study sought to determine whether male participation in the counselling session that utilized a cognitive-behavioural skill approach would significantly impact PMTCT uptake compared to male attendance at antenatal visits only [17].

In four studies, CHWs or patient advocates [7,12,14,26] conducted home visits. These visits were used to communicate information to participants about improving maternal and child health and promoting the utilization of PMTCT services [7,12,14]. The home visits began during antenatal period and continued after delivery. In one study, CHWs followed their clients along the PMTCT continuum of care at their homes and at health centres [26]. In one study [12], CHWs employed cognitive-behavioural intervention strategies in addressing PMTCT uptake.

#### Behavioural interventions

Seven studies tested behavioural interventions [8,11,19,22,25,31,32]. These strategies promote retention and adherence to PMTCT tasks through direct behaviour modification by using techniques such as incentives, reminders and reinforcement. Calling and/or mobile phone texting for education and appointment reminders (six studies) and conditional cash transfer (one study) were the behavioural interventions that were evaluated in these studies. One mobile phone-based SMS reminder study reported using constructs of the Health Belief Model to underpin their intervention [19].

#### Structural and provider-related interventions

Eleven studies evaluated structural interventions [9,10,18,20,24,28–30,33,35,37]. Structural interventions were defined as interventions that aimed to improve PMTCT service delivery by modifying the structural context of a health facility. Eight studies integrated antenatal care (ANC) and HIV treatment services in a single clinic to increase uptake of HIV care and treatment for women and infants [9,10,18,20,24,28,29,35]. One study that integrated HIV and ANC services also trained ANC nurses and midwives, employed lab courier to expedite CD4 count receipt, and conducted home visits and active tracing of mother-infant pairs by lay counsellors [29]. In one study, maternity nurses offered direct accompaniment to the location of exposed infant testing after the birth of a child so as to increase the proportions of HEIs who return for HIV testing and continued monitoring [30]. One trial evaluated targeted training among midwives to increase ARV uptake [33].

### Quality assessment

For each trial quality assessment was guided by Effective Public Health Practice Project criteria [39] (Supplementary file 3). The nature of interventions inhibited blinding of participants and personnel in most studies. Thus, the risk of bias most frequently resulted from lack of blinding (26 out of 32; 81%), loss to follow-up (13 out of 26; 50%) and study design (9 out of 30; 30%). Out of the 34 studies: 11 were cohort studies, nine were cluster randomized trials and randomized trials, seven were pilot studies, three of each were quasi experimental studies and before and after studies and one was stepped-wedge design. Four out of five male partner involvement studies reported low male participation. No studies were excluded on the basis of quality.

### Effects

Details of effect estimates for each intervention are available in Tables 1 to 5.

#### Interventions to increase initiation of ARV/ART in pregnant women

**Figure 2:**
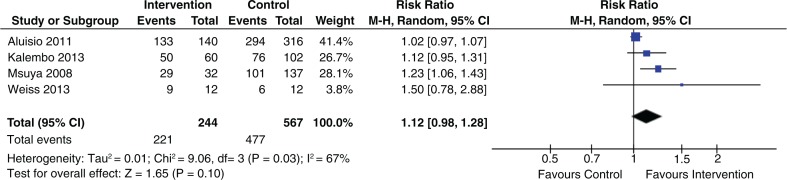
Effect of male partner involvement on ARV/ART initiation.

Of the 21 studies that evaluated intervention to increase ARV/ART uptake in pregnant and breastfeeding women, eight showed statistical significant effects and 13 showed no statistically significant effects (Table 1). Two studies that engaged mentor mothers showed significant benefits [13,36]. Positive results were reported in the only study that used mobile phone text messages [22]. The four studies [17,23,27,34] that evaluated male participation to improve ARV/ART uptake reported non-significant results (pooled RR 1.12; 95% CI 0.98 to 1.28) (Figure 2).

One out of the six studies that evaluated an integrated approach of providing ANC and PMTCT services in a single clinic showed statistically significant benefits [28]. One study [29] evaluated an integrated approach of providing ANC and PMTCT services, trained ANC nurses and midwives, had lab courier to expedite CD4 counts and implemented community-based follow-up of women-infant pairs. This study reported significant results (RR 2.6 95% CI 1.95 to 3.45).

**Figure 3:**
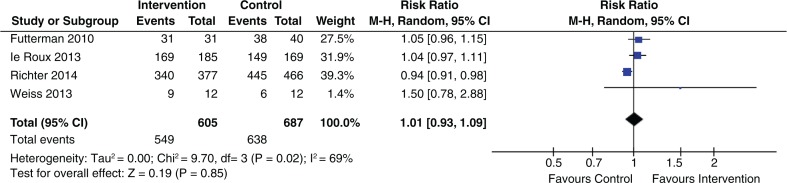
Effect of psychological interventions on ARV/ART initiation.

Five of ten social intervention studies, four of nine structural intervention studies and one behavioural intervention study showed improved uptake of ARV/ART among HIV-positive pregnant women. The four studies [5,12,15,17] that were grounded on psychological interventions to improve ARV/ART uptake reported non-significant results (pooled RR 1.01; 95% CI 0.93 to 1.09) (Figure 3).

#### Interventions to improve retention in PMTCT services

Four of the eight studies that aimed to improve retention in PMTCT services (Table 2) showed statistically significant effects; one study evaluated male partner involvement in PMTCT tasks [27], and the other three were mobile phone-based interventions conducted in Kenya [19,22,25]. The follow-up period ranged from six weeks to eighteen months. The two intervention studies on text messaging reminders and male partner involvement that followed mother-infant pairs for 18 months showed statistically significant benefits (RR 1.91 95% CI 1.71 to 2.15) and (RR 6.44 95% CI 4.93 to 8.41), respectively. Conditional cash transfer showed no statistically significant effects (RR 1.13 95% CI 1.00 to 1.26) [32]. Overall retention rates remained low over time.

#### Interventions to improve uptake of EID

**Figure 4:**
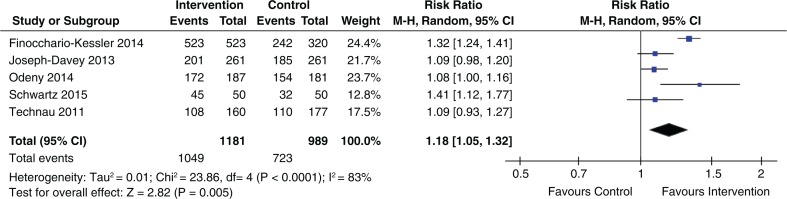
Effect of phone-based reminders on uptake of EID.

Table 3 shows the effect estimates for studies that evaluated interventions to increase uptake of EID. Eleven out of 19 studies that evaluated the initial uptake of EID showed statistically significant benefits, and eight showed no statistically significant benefits. In these studies, the duration of follow-up was six weeks to six months. The pooled effect for five studies evaluating the effect of EID reminders using text messages or phone calls (within six to ten weeks after delivery) showed a statistically significant increase in uptake of infant HIV testing (pooled RR 1.18; 95% CI 1.05 to 1.32) (Figure 4).

Two studies out of the four studies that evaluated peer mentoring outcomes reported an increased number of HEIs tested [36,38]. Out of the seven studies with a home visiting component, six reported statistically significant results [14,16,26,29,36,38]. These studies were very heterogeneous, and no meta-analysis was done.

Five out the six studies [18,20,22,29,36] that evaluated various interventions (peer mentoring, mobile phone texting, integration of ANC and PMTCT services, training of midwives and lab courier services for CD4 count) to improve the uptake of final diagnosis for HEIs, showed statistically significant benefits. The follow-up periods ranged between 9 and 18 months.

**Figure 5:**
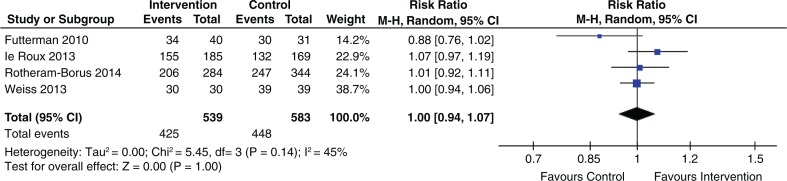
Effect of psychological intervention on uptake of EID.

Four of ten social intervention studies, three of four structural intervention studies and three of five behavioural intervention studies reported increased uptake of EID. Four social intervention studies [6,12,15,17] that were grounded on psychological interventions reported non-significant results (pooled RR 1.00; 95% CI 0.94 to 1.07) (Figure 5).

#### Interventions to increase early ART initiation among HIV-infected infants

Two studies that followed HIV-infected infants until ART initiation showed statistically significant benefits (Table 4). In Finocchario-Kessler *et al*. [22], the time for ART initiation among HIV-infected infants was reduced from 38 to 7 median days. In Kim *et al*. [26], age at ART initiation decreased from median age of 9.1 months to 4.9 months.

#### Infant HIV transmission

**Figure 6:**
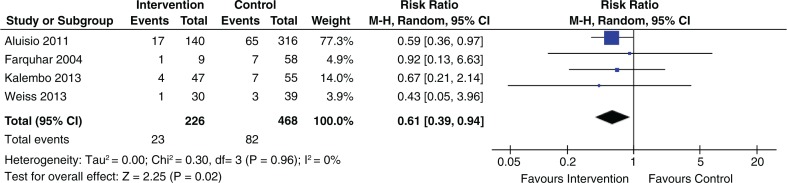
Association of male partner involvement and infant HIV status.

Five out of 13 studies showed an association of reducing vertical HIV transmission. The effect estimates are provided in Table 5. The pooled effect of four studies that evaluated the association of male partner involvement and infant HIV status reported a statistically significant effect (RR 0.61; 95% CI 0.39 to 0.94) (Figure 6).

One study [40] that tested an integrated approach to ANC and PMTCT for mother-infants pair in a single clinic was not associated with reductions in MTCT at six weeks (RR 0.64; 95% CI 0.22 to 1.84) and nine months (RR 0.92; 95% CI 0.55 to 1.53).

Six of 10 social intervention studies, one structural intervention study and one behavioural intervention study did not show an association of reducing infant HIV transmission.

## Discussion

In this systematic review, we identified 34 studies that assessed interventions to improve PMTCT service delivery and promote retention along the PMTCT cascade. We found that male partner involvement in PMTCT reduced infant HIV transmission. Mobile phone-based reminders increased uptake of early infant HIV testing. Home visiting showed some evidence of improving uptake of infant HIV testing. Mobile phone-based reminders showed some evidence of improving retention of mother-infant pairs. Integration of PMTCT services to a single clinic showed no or small benefit in reducing MTCT or increasing ARV/ART uptake among pregnant women. Male partner involvement showed no benefit in increasing ARV/ART uptake among pregnant women. Studies grounded on psychological interventions showed no benefit in increasing ARV/ART uptake or infant HIV testing. Most of the studies were conducted in sub-Saharan Africa.

Behavioural interventions such as mobile phone-based reminders were associated with an 18% increase in the uptake of early infant HIV testing. We think that the evidence supporting this intervention is moderate; it was based on the low rates of retention observed in the intervention and control groups. We, however, suggest that this strategy be used for the wide-scale improvement of the uptake of infant HIV testing and the retention of mother-infant pairs, given the convenience and low cost of delivering SMS [41].

Social interventions, such as male partner involvement in PMTCT, were associated with a 39% reduction in infant HIV transmission. There was, however, no improvement on maternal ARV/ART uptake. The mixed results could probably be an artefact. The quality of support men provided to their HIV-positive partner also varied from attending couple counselling sessions [34] to mitigating the factors that hinder partners’ and infants’ medication adherence (active participation) [17]. Therefore, understanding the aspects of success of male involvement in PMTCT programs warrants further research.

Several factors might have limited other social interventions from showing an effect. For instance, due to the high uptake of PMTCT services in both the intervention and control groups, two studies that engaged peer mentors and one study that engaged CHWs showed no evidence of increasing the number of infants tested for HIV [6,12,15]. Further, due to changes in the national PMTCT guidelines to more effective ARV drugs in both intervention and control group, another study that engaged CHWs showed no evidence in reducing infant HIV transmission [14].

Improved retention did not persist over time, which was illustrated by the low numbers of HEIs whose final HIV status was determined between 9 and 18 months postpartum, as compared to those who had been tested between six weeks and two months [20,22,34,36,40]. These results suggest that it might not be possible to generalize evidence of the effectiveness of interventions to improve retention during the postnatal period from studies of short duration to that of the long term, particularly as several countries are switching to Option B+.

Implementing Option B+ introduces the concept of long-term adherence within PMTCT programs. Structural interventions such as integrating PMTCT services into ANC may provide continuous care at a single clinic and facilitate the tracking of pregnant and/or breastfeeding mothers who have missed their appointments [42]. Nevertheless, the studies reviewed and two previous systematic reviews did not find evidence of significant effectiveness on the uptake of PMTCT services [43,44]. Several reasons for the scant evidence of PMTCT service integration on improving service delivery have been identified, including poor infrastructure, shortage of trained health care workers, low rates of client retention, the small number of study sites and poor service data collection [9,10,18,24].

Only one pilot study [8] that evaluated mobile phone-based reminders was implemented in a facility that offered Option B+. Ongoing and planned clinical trials to address utilization and retention challenges in Option B+ include point of care provision of CD4 tests, engaging mentor mothers, participating in mother support groups, sending SMS reminders for clinic appointments and offering facility-based and community-based peer support [45–49].

### Limitations

We were unable to carry out a subgroup analysis of the primary groupings of the interventions which included social, behavioural and structural, because interventions differed in terms of sample size, timing, setting, personnel, duration and delivery mode. Because we did not restrict our searches to studies with a minimum sample size or a minimum duration of follow-up, the studies included may have been limited in statistical power [50]. We found limited evidence to support the effectiveness of interventions aimed at improving infant ART uptake as literature was limited. Although we conducted an extensive search of several databases and included all types of study design, the studies included in this systematic review were mainly from sub-Saharan Africa.

## Conclusions

In this systematic review of interventions aimed at identifying interventions that improved PMTCT service delivery, we identified two interventions with significant effects: mobile phone-based reminders increased the uptake of early infant HIV testing and male partner involvement in PMTCT was associated with reductions in infant HIV transmission. Given the rapid expansion of PMCT programs, future studies should determine the long-term effects of these interventions in improving adherence and retention in the several steps of the PMTCT cascade.

## Supplementary Material

A systematic review of interventions to improve prevention of mother-to-child HIV transmission service delivery and promote retention
