# Supplementary material for: A systematic review of interventions to improve prevention of mother-to-child HIV transmission service delivery and promote retention
Source: J Int AIDS Soc. 2016 Apr 6;19(1):20309. doi: 10.7448/IAS.19.1.20309 (PMC4824870; doi:10.7448/IAS.19.1.20309)
Supplement: A systematic review of interventions to improve prevention of mother-to-child HIV transmission service delivery and promote retention [file JIAS-19-20309-s001.pdf]

Additional file 1: Search Strategies

PubMed search strategy

	Domain	Search terms
1	HIV	HIV OR human immunodeficiency virus OR AIDS OR acquired immune deficiency syndrome
		**AND**
2	ART	ART OR antiretroviral* OR anti-retroviral* OR HAART OR highly active antiretroviral therapy OR cART OR combined antiretroviral therapy
		**AND**
3	Pregnant	matern* OR pregnan* OR expectant mother OR expectant mothers OR delivery OR postpartum period OR post-partum period OR breast feeding OR breastfeeding OR lactat*
		**AND**
4	PMTCT	antenatal HIV test OR maternal HIV test OR infant HIV test OR infant HIV diagnosis OR option A OR option B OR option B+ OR nevirapine OR mother-to-child transmission OR MTCT OR prevention of mother-to-child transmission OR PMTCT OR PMTCT cascade
		**AND**
5	Utilisation	increas* OR enhanc*OR improv* OR utiliz* OR utilis* OR uptake OR access* OR optimiz* OR optimis* OR retention

Final search: (#1 AND #2 AND (#3 OR #4) AND #5)

Embase search strategy

	Domain	Search terms
1	HIV	HIV OR human immunodeficiency virus OR AIDS OR acquired immune deficiency syndrome
		**AND**
2	ART	ART OR antiretroviral OR anti-retroviral OR HAART OR highly active antiretroviral therapy OR cART OR combined antiretroviral therapy
		**AND**
3	Pregnant	matern* OR pregnan* OR expectant mother OR expectant mothers OR delivery OR postpartum period OR post-partum period OR breast feeding OR breastfeeding OR lactat*
		**AND**
4	PMTCT	antenatal HIV test OR maternal HIV test OR infant HIV test OR infant HIV diagnosis OR option A OR option B OR option B+ OR nevirapine OR exp vertical transmission/
		**AND**
5	Utilisation	increas* OR enhanc*OR improv* OR utiliz* OR utilis* OR uptake OR access* OR optimiz* OR optimis* OR retention

Final search: (#1 AND #2 AND (#3 OR #4) AND #5)

Web of Science search strategy

	Domain	Search terms
1	HIV	"HIV" OR "human immunodeficiency virus" OR "AIDS" OR "acquired immune deficiency syndrome"

		**AND**
2	ART	"ART" OR antiretroviral* OR anti-retroviral* OR "HAART" OR "highly active antiretroviral therapy" OR "cART" OR "combined antiretroviral therapy"
		**AND**
3	Pregnant	matern* OR pregnan* OR "expectant mother" OR "expectant mothers" OR "delivery" OR "postpartum period" OR "post-partum period" OR "breast feeding" OR "breastfeeding" OR lactat*
		**AND**
4	PMTCT	"antenatal HIV test" OR " maternal HIV test" OR "infant HIV test" OR "infant HIV diagnosis" OR "option A" OR "option B" OR "option B+" OR "nevirapine" OR "mother-to-child transmission" OR "MTCT" OR "prevention of mother-to-child transmission" OR "PMTCT" OR "PMTCT cascade"
		**AND**
5	Utilisation	increas* OR enhanc*OR improv* OR utiliz* OR utilis* OR uptake OR access* OR optimiz* OR optimis* OR "retention"

Final search: (#1 AND #2 AND (#3 OR #4) AND #5)

Additional File 2: Studies included in the review

Intervention category	Author Year	Study period	Study country	Study design	Aims	Intervention
Social	Aluisio 2011 [23]	1999-2005	Kenya	Prospective cohort	To investigate the relationship between male partner involvement in PMTCT services and infant HIV acquisition	Enrolled partners received counselling on vertical transmission and prevention methods from trained study personnel in the antenatal clinic.
	Farquhar 2004 [21]	2001-2002	Kenya	Prospective cohort	To determine effect of partner involvement and couple counseling on uptake of PMTCT interventions.	Couples received behavioural interventions to prevent mother-to-child transmission.
	Kalembo 2013 [27]	2004-2006	Malawi	Retrospective Cohort	To examine the association between male partner involvement and the uptake of PMTCT interventions	Couples received behavioural interventions to prevent mother-to-child transmission.
	Msuya 2008 [34]	2002-2004	Tanzania	Prospective cohort	To describe effect of partner participation on uptake of HIV perinatal interventions	Couples received behavioural interventions to prevent mother-to-child transmission.
	Weiss 2013 [17]	2010-2011	South Africa	Pilot study (Randomised controlled trial)	To determine whether male participation in the intervention would significantly impact PMTCT uptake.	Four successive weekly sessions that utilised a cognitive–behavioural skill training approach to improve adherence to ARV/ART.
	Baek 2007 [13]	2005-2006	South Africa	Pre-post, quasi-experimental (Cross-sectional surveys)	To investigate whether mentor mothers increase utilisation of PMTCT services	A peer support intervention that provided education and psychosocial support to HIV-positive pregnant women and new mothers and followed up HIV positive mothers and their babies at home and the health facility.
	ENHAT-CS 2014 [36]	2008-2011	Ethiopia	Retrospective cohort	To investigate the effect of mentor mothers on uptake of EID and HIV infection rates.	Individual and mother support groups behavioural sessions were delivered by peer mentors. Clients were also followed at home and at the health facility.
	Futterman 2010 [15]	2006-2007	South Africa	Pilot study (two different clinics)	To examine the effectiveness of mentoring mothers and a cognitive-behavioural intervention to PMTCT services	Eight sessions of a cognitive-behavioural intervention delivered by peer mentors that focused on, prevention of HIV transmission, adherence to pre- and post-natal baby treatment, and uptake of EID.

Intervention category	Author Year	Study period	Study country	Study design	Aims	Intervention
	Richter 2014 [5]	2008-2010	South Africa	Cluster randomised trial	To assess efficacy of peer mentoring support from pregnancy to 1.5 months post-birth.	An eight-session intervention (four antenatal sessions, four postnatal sessions) delivered by peer mentors that supported women living with HIV (WLH) through pregnancy and early motherhood, by encouraging them to adhere to PMTCT tasks.
	Rotheram-Borus 2014 [6]	2008-2010	South Africa	Cluster randomised trial	To evaluate the effect of clinic-based peer mentoring on maternal and infant well-being among WLH from pregnancy through the infant's first year of life.	Four antenatal and four postnatal sessions of a cognitive-behavioural intervention led by HIV-positive peer mentors that focused on supporting WLH to cope with their HIV status and adhere to PMTCT tasks.
	Le Roux 2013 [12]	2009-2010	South Africa	Cluster randomised trial	To evaluate the effect of home visits by CHWs on maternal and infant well-being from pregnancy through the first six months of life for WLH	A home-visiting intervention by community health workers grounded in cognitive-behavioural approach involving an average of six antenatal and five postnatal visits that focused on prevention of HIV transmission, and completion of PMTCT tasks.
	Tomlinson 2014 [14]	2008-2010	South Africa	Cluster randomised trial	To assess the effect of CHWs home visits to pregnant and post-natal women on infant HIV-free survival	A home-visiting intervention delivered by CHWs involving two pregnancy visits and five post-natal visits that focused on, promoting retention and adherence to PMTCT tasks.
	Kim 2012 [26]	2009-2011	Malawi	Pilot study (Pre-intervention & intervention study)	To evaluate the effect of CHWs to utilisation of PMTCT services and patient retention	CHWs followed their clients at their homes and at health centres, from initial diagnosis up until confirmation of definitive HIV-uninfected status after cessation of breastfeeding or successful ART initiation for HIV-infected infants.
	Rundare 2012 [7]	2011	South Africa	Before and after design	To evaluate the effect of community-based adherence support (CBAS) on uptake of 6 week EID and HIV transmission.	A home visiting intervention provided by patient advocates during antenatal, delivery and postnatal periods by providing psychosocial support and encouraging mothers to adhere to PMTCT tasks.

Intervention category	Author Year	Study period	Study country	Study design	Aims	Intervention
	Shroufi 2013 [38]	2011	Zimbabwe	Retrospective cohort	To investigate whether mentor mothers increase utilisation of PMTCT services	A peer support intervention that provided education and psychosocial support to HIV-positive pregnant women and new mothers and followed up mothers and babies at home and the health facility to ensure they received appropriate medical care after delivery.
Behavioural	Finocchario-Kessler 2014 [22]	2010-2012	Kenya	Observational pilot study	To evaluate the impact of text messaging to improve retention in EID cascade of care.	Text messages were sent to HIV-infected mothers' mobile phones when infant HIV test results were available.
	Joseph-Davey 2013 [31]	2011	Mozambique	Randomised controlled trial	To investigate the efficacy of sending regular SMS reminders and educational messages on improving retention in PMTCT care	HIV pregnant women received appointment reminders and educational messages via SMS.
	Kebaya 2015 [25]	NR	Kenya	Open label randomised controlled trial	To compare self-reported adherence to infant nevirapine (NVP) prophylaxis and retention in care over 10 weeks	Mothers of HIV-exposed infants received 2-weekly mobiles phone calls.
	Odeny 2014 [19]	2012-2013	Kenya	Randomised controlled trial	To investigate the effect of interactive text messaging on clinic retention and uptake of EID	Text messages were developed based on constructs of the health belief model. The SMS group received up to eight text messages before delivery and six messages post-partum.
	Schwartz 2015 [8]	2013	South Africa	Pilot study (Pre-intervention and intervention design)	To assess the acceptability and feasibility of a cell phone based case manager intervention targeting HIV-infected pregnant women on HAART	Pregnant women ≥36 weeks gestation attending antenatal care and receiving HAART through the Option B+ program received text messages and telephone calls from a case manager through 6 weeks postpartum.
	Technau 2011 [11]	NR	South Africa	Observational cohort in two clinics	To test whether mobile phone text messages (SMSs) improve infant follow-up rates	Appointment reminders for infant PMTCT medication and infant HIV testing were sent via SMS.

Intervention category	Author Year	Study period	Study country	Study design	Aims	Intervention
	Yotebieng 2015 [32]	2013-2014	Democratic Republic of Congo	Randomised controlled trial	To determine whether small, increasing cash payments conditioned on attending scheduled clinic visits and receiving proposed services can increase the proportion of HIV-infected pregnant women who attend PMTCT visits and adhere to available PMTCT services through six weeks postpartum.	Newly diagnosed HIV-infected women, ≤32 weeks pregnant were given financial incentive ($5 plus $1 increment at each subsequent visit) to attend regular clinic visits and receive PMTCT care.
Structural	Kieffer 2011 [33]	2008-2009	Swaziland	Quasi-experimental	To evaluate the effect of targeted training for maternity nurses on patients' uptake of ARV prophylaxis before delivery	Targeted on-site training of nurse-midwives on PMTCT tasks.
	Ciampa 2011 [30]	2009-2010	Mozambique	Pilot study (Retrospective cohort)	To investigate the effect of enhanced referral to improve uptake of EID	HIV-infected mothers were directly accompanied by a maternity nurse before discharge to the location of EID services within the hospital grounds.
	Killam 2010 [28]	2007-2008	Zambia	Stepped-wedge design	To evaluate whether providing ART integrated in ANC increases initiation of ART	Providing ART integrated in ANC.
	Stinson 2010 [9]	2005	South Africa	Retrospective cohort	To evaluate the effect of referral to another clinic or integration of PMTCT services in one clinic on ART initiation.	Integrated model in which women were able to initiate ART within the ANC on one specific day of the week when the outreach doctors were on site.
	Stinson 2013 [10]	2008	South Africa	Retrospective cohort	To evaluate the effect of referral to another clinic or integration of PMTCT services in one clinic on ART initiation.	Integrated model in which women were able to initiate ART within the ANC on one specific day of the week when obstetricians with an HIV behavioral on were on site.
	Tsague 2010 [35]	2007-2008	Rwanda	Before and after study	To compare the performance of sites providing PMTCT as stand-alone service versus sites providing PMTCT as well as ART.	Integrating PMTCT and ART services on the same premises.

Intervention category	Author Year	Study period	Study country	Study design	Aims	Intervention
	Turan 2015 [18]	2009-2011	Kenya	Cluster randomised trial	To assess whether integrating ANC and HIV treatment services for pregnant women in a single clinic improves PMTCT utilisation.	Integrating ANC and HIV treatment services in a single clinic.
	Van't Hoog 2005 [24]	2001-2003	Kenya	Pilot project (Before and after design)	To evaluate whether integration of PMTCT services into ANC increases uptake.	Integration of PMTCT services into ANC.
	Ezeanolue 2015 [37]	2005-2012	U.S. A	Before and after study	To investigate an integrated approach between public health department, primary care and medical care on reducing or eliminating MTCT of HIV.	Enhanced referral to an adult HIV specialist, obstetrician, paediatrician and public health department.
Structural and social	Ong'ech 2012 [20]	2008 - 2010	Kenya	Observational prospective cohort study	To evaluate effectiveness of integrated MCH model compared with CCC model on retention.	PMTCT services were provided within MCH clinics or in specialised HIV CCCs. Linkages between MCH (intervention) and CCC (control) were facilitated by peer counsellors, who provided ongoing support to women and escorted them between the 2 clinics.
	Herlihy 2015 [29]	2011-2013	Zambia	Quasi-experimental design with pre/post-intervention	To evaluate whether integration of PMTCT and ANC services improves ART uptake and streamlines ART initiation.	Integrating ANC and HIV treatment services in a single clinic, training of ANC nurses and midwives, employed lab courier to expedite CD4 count receipt, and home visits and active tracing was done by lay counsellors.
Social and behavioural	Besser 2010 [16]	NR	South Africa	Prospective cohort	To increase uptake of EID	A peer support intervention and phone calls were made to mothers who had not brought their infants for testing at 8 weeks. Further, home visits (by mentor mothers) were conducted among women who had not taken their infants for EID two weeks later.

Additional file 3: Quality assessment summary table

Intervention category	Summary of intervention type	Author Year	Selection bias	Study design	Confounders affected PMTCT outcomes	Blinded assessment of study outcomes	Data collection methods	Withdrawal and dropouts	Integrity of intervention[a]
Social	Male partner involvement	Aluisio 2011 [23]	Weak	Moderate	Strong	Weak	Strong	Strong	Moderate
		Farquhar 2004 [21]	Weak	Moderate	Strong	Weak	Strong	Weak	Moderate
		Kalembo 2013 [27]	Moderate	Moderate	Strong	Weak	Moderate	Weak	Moderate
		Msuya 2008 [34]	Weak	Moderate	Strong	Weak	Strong	Weak	Moderate
		Weiss 2013 [17]	Strong	Strong	Strong	Moderate	Strong	Strong	Strong
	Peer mentoring	Baek 2007 [13]	Strong	Moderate	Strong	Weak	Strong	Not applicable[b]	Moderate
		ENHAT-CS 2014 [36]	Moderate	Moderate	Strong	Weak	Moderate	Strong	Moderate
		Futterman 2010 [15]	Weak	Weak	Moderate	Weak	Moderate	Weak	Strong
		Richter 2014 [5]	Moderate	Moderate	Strong	Weak	Strong	Weak	Strong
		Rotheram-Borus 2014 [6]	Moderate	Strong	Strong	Weak	Strong	Weak	Strong
		Shroufi 2013 [38]	Moderate	Moderate	NR	Weak	Strong	Strong	Strong
	CHWs	Kim 2012 [26]	Strong	Weak	Moderate	Weak	Moderate	Strong	Strong
		Le Roux 2013 [12]	Strong	Strong	Strong	Strong	Strong	Strong	Strong
		Tomlinson 2014 [14]	Strong	Strong	Weak	Moderate	Moderate	Strong	Strong
	Patient advocates	Rundare 2012 [7]	Moderate	Weak	Strong	Weak	Strong	Weak	Moderate
Behavioural	Mobile-phone text messages	Finocchario-Kessler 2014 [22]	Moderate	Weak	Strong	Weak	Strong	Strong	Strong
		Joseph-Davey 2013 [31]	Moderate	Strong	Strong	Strong	Strong	Strong	Moderate
		Odeny 2014 [19]	Strong	Strong	Strong	Strong	Strong	Weak	Strong
		Technau 2011 [11]	NR[a]	Strong	NR[a]	NR[a]	Strong	Moderate	Strong
	Mobile-phone calls	Kebaya 2015 [25]	Strong	Strong	Strong	Weak	Strong	Weak	Strong
	Calls and mobile-phone text message	Schwartz 2015 [8]	Moderate	Weak	Strong	Weak	Strong	Moderate	Strong
	Conditional cash transfer	Yotebieng 2015 [32]	Strong	Strong	NR[a]	Strong	Strong	Moderate	Strong
Structural	Enhanced referral	Ciampa 2011 [30]	Moderate	Moderate	Strong	Weak	Moderate	Weak	Moderate
	Training of midwives	Kieffer 2011 [33]	Strong	Strong	Strong	Weak	Strong	Not applicable[c]	Strong
	Integration of PMTCT into routine pregnancy and infant care	Killam 2010 [28]	Strong	Strong	Strong	Weak	Strong	Strong	Strong
		Stinson 2010 [9]	Strong	Moderate	Strong	Weak	Moderate	NR	Moderate
		Stinson 2013 [10]	Strong	Moderate	Strong	Weak	Moderate	NR	Moderate
		Tsague 2010 [35]	Moderate	Weak	Weak	Weak	Strong	NR	Strong
		Turan 2015 [18]	Strong	Strong	Moderate	Moderate	Strong	Moderate	Moderate

Intervention category	Summary of intervention type	Author Year	Selection bias	Study design	Confounders affected PMTCT outcomes	Blinded assessment of study outcomes	Data collection methods	Withdrawal and dropouts	Integrity of intervention[a]
		van't Hoog 2005 [24]	Strong	Weak	Strong	Weak	Strong	Weak	Strong
	Integration of public health services into clinical care for HIV- infected pregnant women and HEI	Ezeanolue 2015 [37]	Weak	Weak	Weak	Weak	Strong	Strong	Moderate
Social and behavioural	Peer mentoring and mobile-phone calls	Besser 2010 [16]	Strong	Moderate	NR[a]	Weak	Strong	Weak	Strong
Structural and social	Integration of PMTCT into routine pregnancy and infant care and use of peer counsellors	Ong'ech 2012 [20]	Strong	Weak	Moderate	Weak	Strong	Weak	Strong
	Integration of PMTCT and ANC services, lab courier system for CD4 counts and use of lay counsellors	Herlihy 2015 [29]	Moderate	Strong	Moderate	Weak	Weak	Moderate	Strong

[a] Non-uniformity between intervention and control groups [b] Cross-sectional survey [c] Ingestion of birth NVP syrup assessed in the medical records [d] Conference abstract NR-not reported

Weak (<60% comparable between arms), moderate (60-79% comparable between arms), and strong (>80% comparable between arms)
